# A click-based electrocorticographic brain-computer interface enables long-term high-performance switch-scan spelling

**DOI:** 10.21203/rs.3.rs-3158792/v1

**Published:** 2023-09-25

**Authors:** Nathan Crone, Daniel Candrea, Samyak Shah, Shiyu Luo, Miguel Angrick, Qinwan Rabbani, Christopher Coogan, Griffn Milsap, Kevin Nathan, Brock Wester, William Anderson, Kathryn Rosenblatt, Lora Clawson, Nicholas Maragakis, Mariska Vansteensel, Francesco Tenore, Nick Ramsey, Matthew Fifer, Alpa Uchil

**Affiliations:** Johns Hopkins Hospital; Johns Hopkins University; Johns Hopkins Hospital; Johns Hopkins University; Johns Hopkins Hospital; Johns Hopkins University; Johns Hopkins Hospital; Johns Hopkins University Applied Physics Lab; Johns Hopkins Hospital; Johns Hopkins University Applied Physics Lab; Johns Hopkins University School of Medicine; Johns Hopkins Hospital; Johns Hopkins Hospital; Johns Hopkins Hospital; UMC Utrecht Brain Center; Johns Hopkins University Applied Physics Lab; UMC Utrecht Brain Center; JHU Applied Physics Laboratory; JHU Applied Physics Laboratory

## Abstract

**Background:**

Brain-computer interfaces (BCIs) can restore communication in movement- and/or speech-impaired individuals by enabling neural control of computer typing applications. Single command “click” decoders provide a basic yet highly functional capability.

**Methods:**

We sought to test the performance and long-term stability of click-decoding using a chronically implanted high density electrocorticographic (ECoG) BCI with coverage of the sensorimotor cortex in a human clinical trial participant (ClinicalTrials.gov, NCT03567213) with amyotrophic lateral sclerosis (ALS). We trained the participant’s click decoder using a small amount of training data (< 44 minutes across four days) collected up to 21 days prior to BCI use, and then tested it over a period of 90 days without any retraining or updating.

**Results:**

Using this click decoder to navigate a switch-scanning spelling interface, the study participant was able to maintain a median spelling rate of 10.2 characters per min. Though a transient reduction in signal power modulation interrupted testing with this fixed model, a new click decoder achieved comparable performance despite being trained with even less data (< 15 min, within one day).

**Conclusion:**

These results demonstrate that a click decoder can be trained with a small ECoG dataset while retaining robust performance for extended periods, providing functional text-based communication to BCI users.

## Plain Language Summary

People living with amyotrophic lateral sclerosis (ALS) struggle to communicate with family and caregivers due to progressive muscle weakness. This study investigated whether the brain signals of a participant with ALS could be used to control a spelling application. Specifically, when the participant attempted to make a fist, a computer algorithm detected increased neural activity from electrodes implanted on the surface of his brain, and thereby generated a mouse-click. The participant used these self-generated clicks to select letters or words from a spelling application to type sentences. Our algorithm was trained using less than one hour’s worth of recorded brain signals and then performed reliably for a period of three months. This approach can potentially be used to restore communication to other severely paralyzed individuals over an extended period of time and after only a short training period.

## Introduction

Brain-computer interfaces (BCIs) can allow individuals with a variety of motor impairments to control assistive devices using their neural signals^1–11^. In particular, implantable BCIs have the potential to provide higher performance compared to non-invasive BCIs and may provide round-the-clock availability. These capabilities are derived either from single neuron activity recorded by microelectrode arrays (MEAs), or from neural population activity recorded by macroelectrodes (typically consisting of electrocorticographic (ECoG) arrays on the cortical surface)^12^. Although sophisticated capabilities and high performance of MEA BCIs have been reported, their use outside of research environments has been limited due to varying degrees of long-term signal attrition^13,14^ and day-to-day instability in decoding models trained on single neuron activity, often requiring frequent recalibration^15^. On the other hand, extensive safety and efficacy data from the use of chronic ECoG recordings for epilepsy management^16^ suggests that ECoG implants have the potential to deliver greater long-term signal stability. However, the utility of ECoG for chronically implanted BCIs has only been tested in a few participants.

In the first clinical trial of a chronic ECoG BCI^1^, a participant with quadriplegia and anarthria due to amyotrophic lateral sclerosis (ALS) attempted hand movements to generate “brain clicks”, in turn controlling a switch-scanning spelling application. These brain clicks were detected as spectral changes in ECoG signals recorded from a single pair of electrodes on the surface of hand area of contralateral motor cortex. Though the participant used these brain clicks to communicate in her daily life for more than 3 years^17^, several months of data collection were necessary for parameter optimization. In a separate clinical trial^4,18^, participants with severe upper limb paralysis due to ALS or primary lateral sclerosis were implanted with an endovascular stent-electrode array and required 1–12 sessions of training with their brain click BCI before long-term use^4^. However, due to the location of electrodes in the superior sagittal sinus, the participants triggered brain clicks with attempted foot movements, which may not be intuitive for computer control. Moreover, device limitations in both clinical trials may have constrained brain click speed and overall performance of the BCIs. Vansteensel et al. reported 87–91% click accuracy (comprised of correctly detected and withheld clicks) with a 1 s latency^1^ while Mitchell et al. reported ~ 82% accuracy with a 0.9 s latency or a 97% accuracy with a 2.5 s latency^4^.

In this study, we tested whether improved click performance could be achieved using high density ECoG recordings from sensorimotor cortex. We implanted two 8 × 8 ECoG grids (4 mm pitch, PMT Corp., Chanhassen, MN) over left hand and face cortical regions in a clinical trial participant with ALS (ClinicalTrials.gov, NCT03567213). The participant generated clicks using the implanted BCI to spell sentences at a significantly improved spelling rate compared to prior brain click work using a switchscanning paradigm^1^. Moreover, the participant achieved high click-detection accuracies with low falsepositive rates and low latencies from attempted movement onset to click. We found that a fixed EcoGbased click decoder trained on a limited dataset maintained high performance over a period of several months without requiring re-training or daily model adaptation. Finally, offline analysis suggested that similar performance is achievable with a smaller number of ECoG electrodes over only the cortical handknob region.

## Methods

### Clinical trial

This study was performed as part of the CortiCom clinical trial (Clinicaltrials.gov Identifier: NCT03567213), a phase I early feasibility study of the safety and preliminary efficacy of an implantable ECoG BCI. Due to the exploratory nature of this study and the limited number of participants, the primary outcomes of the trial were stated in general terms (Supplementary Note 1) and were designed to gather preliminary data on: 1) the safety of the implanted device, 2) the recording viability of the implanted device, and 3) BCI functionality enabled by the implanted device using a variety of strategies. No methods or statistical analysis plans were predefined for assessing these outcomes given their exploratory nature and the limited number of participants. Results related to the first two primary outcome variables, though necessarily provisional as they are drawn from only one participant, are reported in Supplementary Notes 2 and 3 respectively. Results related to BCI functionality, also necessarily provisional and exploratory (Supplementary Note 4), are addressed within the subsequent methodology and results, which nevertheless employed rigorous analyses and statistics.

The study protocol can be found as an additional supplemental file. The study protocol was reviewed and approved by Johns Hopkins University Institutional Review Board and by the US Food and Drug Administration (FDA) under an investigational device exemption (IDE).

### Participant

All results reported here were based on data from the first and only participant to date in the CortiCom trial. The participant gave written consent after being informed of the nature of the research and implant related risks. To date this participant has had no serious or device-related adverse events, and thus the primary outcome of the CortiCom trial has been successful. The secondary outcomes of the CortiCom trial are reported, in part, here; specifically, our success rate and latency are reported in terms of click detection accuracy and time from attempted movement onset to click.

The participant was a right-handed man who was 61 years old at the time of implant in July 2022 and diagnosed with ALS roughly 8 years prior. Due to bulbar dysfunction, the participant had severe dysphagia and progressive dysarthria. This was accompanied by progressive dyspnea. The participant could still produce overt speech, but slowly and with limited intelligibility. He had experienced progressive weakness in his upper limbs such that he is incapable of performing activities of daily living without assistance; his lower limbs are less affected.

### Neural implant

**Figure 1:**
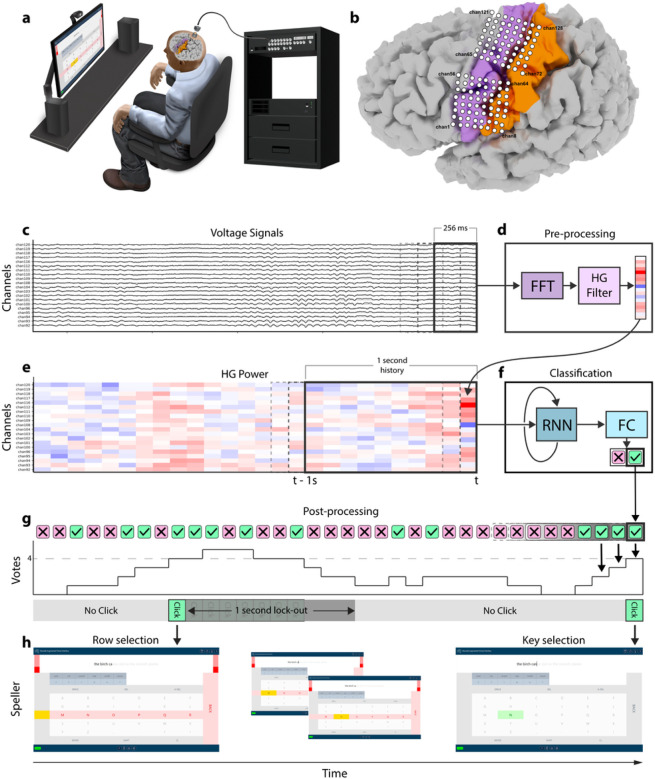
Real-time decoding pipeline. **(a)** The participant was seated upright with his forearms on the armrests of a chair facing a computer monitor where the switch-scanning speller application was displayed. **(b)** Position of both 64-electrode grids overlayed on the left cortical surface of the participant’s brain. The dorsal and ventral grids primarily covered cortical upper limb and face regions respectively. The electrodes are numbered in increasing order from left to right and from bottom to top. Magenta: pre-central gyrus; Orange: post-central gyrus. **(c)** ECoG voltage signals were streamed in 100 ms packets to update a 256 ms running buffer for real-time spectral pre-processing. A sample of signals from 20 channels is shown. **(d)** A Fast Fourier Transform filter was used to compute the spectral power of the 256 ms buffer, from which the HG log-power (110–170 Hz) was placed into a 1 s running buffer (10 feature vectors). **(e)** The running buffer was then used as time history for the recurrent neural network (RNN). **(f)** An RNN-FC (RNN-fully connected) network then predicted rest or grasp every 100 ms depending on the higher output probability. **(g)** Each classification result was stored as a vote in a 7-vote running buffer such that the number of grasp votes had to surpass a predetermined voting threshold (4-vote threshold shown) to initiate a click. A lock-out period of 1 s immediately followed every detected click to prohibit multiple clicks from occurring during the same attempted movement. **(h)** Once a click was detected, the switch scanning speller selected the highlighted row or element within that row. Two clicks were necessary to type a letter or autocomplete a word.

The CortiCom study device was composed of two 8×8 subdural ECoG grids manufactured by PMT Corporation (Chanhassen, MN), which were connected to a percutaneous 128-channel Neuroport pedestal manufactured by Blackrock Neurotech Corporation (Salt Lake City, UT). Final assembly and sterilization of the study device was performed by Blackrock Neurotech. Both subdural grids consisted of soft silastic sheets embedded with platinum-iridium disc electrodes (0.76 mm thickness, 2-mm diameter exposed surface) with 4 mm center-to-center spacing and a total surface area of 12.11 cm^2^ (36.6 mm × 33.1 mm). The device included two reference wires, which were exposed to match the recording surface area of the ECoG electrodes. During all recordings with the study device, the Neuroport pedestal was coupled to a small (24.9 mm × 17.7 mm × 17.9 mm) external device (Neuroplex-E; Blackrock NeurotechCorp.) for signal amplification, digitization, and digital transmission via a mini-HDMI cable to the Neuroport Biopotential System (Blackrock Neurotech Corp.) (Fig. 1a).

The two electrode grids of the study device were surgically implanted subdurally, over sensorimotor cortex representations for speech and upper extremity movements in the left hemisphere. Implantation was performed via craniotomy under monitored anesthesia care with local anesthesia and sedation tailored to intraoperative task participation. There were no surgical complications or surgically related adverse events. The locations of targeted cortical representations were estimated prior to implantation using anatomical landmarks from a pre-operative structural MRI, functional MRI, and somatosensory evoked potentials. The locations of the subdural grids with respect to surface gyral anatomy were confirmed after implantation by co-registering a post-operative high-resolution CT with a pre-operative high-resolution MRI using Freesurfer^19^ (Fig. 1b).

### Testing and calibration

At the beginning of each session, a 60 second calibration period was recorded, during which the participant was instructed to sit still and quiet with his eyes open and visually fixated on a computer monitor. For each channel, we then computed the mean and standard deviation of the spectral-temporal log-powers for each frequency bin. These estimates of resting baseline cortical activity were subsequently used for normalization of power estimates during model training and BCI operation.

### Training task

**Figure 2:**
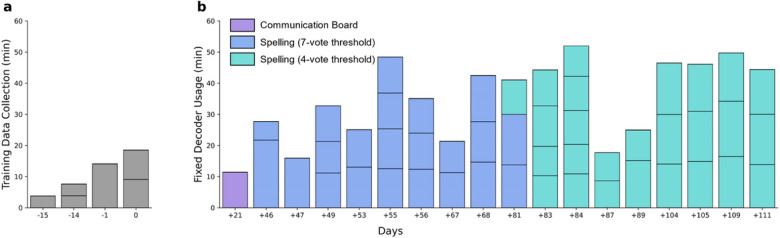
Long-term use of a fixed click detector. **(a)** Training data was collected during 4 sessions that occurred within a period of 15 days. For each day, each sub-bar represents a separate block of training data collection (6 training blocks total). **(b)** Using the fixed decoder, one block of switch-scanning with the communication board was performed +21 days post-training data collection (purple). From Day +46 to Day +81, the fixed decoder was used for switch-scan spelling with a 7-vote threshold (blue). From Day +81 to Day +111, the fixed decoder was used for switch-scan spelling with a 4-vote threshold (teal). For each day, each sub-bar represents a separate spelling block of 3–4 sentences. The horizontal axis spanning both **(a)** and **(b)** represents the number of days relative to the last day of training data collection (Day 0).

Training data was collected across four sessions (six training blocks in total) spanning 15 days (Fig. 2a). For each block, the participant was instructed to attempt a brief grasp with his right hand (i.e., contralateral to the implanted arrays) in response to visual cues (Supplementary Fig. 1). Due to the participant’s severe upper extremity impairments, his attempted movements primarily involved flexion of the middle and ring fingers. After each attempt, the participant released his grasp and passively allowed his hand to return to its resting position hanging from the wrist at the end of his chair’s armrest.

Each trial of the training task consisted of a single 100 ms “Go” stimulus prompting the participant to attempt a grasp, followed by an interstimulus interval (ISI), during which the participant remained still and fixated his gaze on a crosshair in the center of the monitor. Previous experiments using longer cues had resulted in more variable response latencies and durations. The length of each ISI was randomly chosen to vary uniformly between a lower and upper bound to reduce anticipatory behavior. The experimental parameters across all training sessions are shown in Supplementary Table 1. In total, almost 44 min of data (260 trials) was collected for model training.

### Data collection

Neural signals were recorded by the Neuroport system at a sampling rate of 1 kHz. BCI2000 was used to present stimuli during training blocks and to store the data for offline analysis^20^. Video of the participant’s right hand (i.e., which was overtly attempting grasp movements) and the monitor displaying the spelling application was recorded at 30 frames per second (FPS) during all spelling sessions except the last two (at 60 FPS). A 150 ms synchronization audio cue was played at the beginning of each spelling block (*see* Real-Time Switch-Scanning) so that the audio recorded by the Neuroport biopotential system’s analog input could be used offline to synchronize the video frames with the neural data. A pose estimation algorithm^21^ was applied offline to the hand video to infer the horizontal and vertical positions of 21 hand and finger landmarks within each video frame. The horizontal coordinates of the metacarpalphalangeal (MCP) joint landmarks for the first and fifth digits were used to normalize horizontal positions of all landmarks, while the MCP and fingertip coordinates of the same digits were used to normalize vertical positions.

### Feature extraction and label assignment

For each of the 128 recording channels, we used a Fast Fourier Transform (FFT) filter to compute the spectral power of 256 ms windows shifted by 100 ms increments. The spectral power in each frequency bin was log-transformed and normalized to the corresponding calibration statistics. We summed the spectral power in the frequency band between 110 and 170 Hz to compute our high-gamma (HG) power. We chose this lower bound of the frequency band because post-movement low frequency activity sometimes extended to 100 Hz in several channels (Supplementary Fig. 2). This resulted in a 128-channel feature vector that was used in subsequent model training.

After computing each channel’s trial-aligned HG power (−1 s to 2.5 s post-cue), we accounted for the intertrial variability due to reaction delay by re-aligning each trial’s HG power using a subset of highly activated channels^22^. This resulted in generally increased HG power correlations between trials (Supplementary Figs. 3–5). We visually determined the onset and offset of the re-aligned trial-averaged HG power from the channels used for re-alignment (Supplementary Fig. 6). The average neural activity onset and offset were manually estimated from the aligned neural data to be roughly 0.2 s and 1.2 s post-cue, respectively, with neural activity more clearly differentiating from rest activity starting at 0.3 s post-cue and ending at 1.1 s post-cue. We consequently assigned grasp labels to ECoG feature vectors falling between 0.3 s and 1.1 s post-cue for each trial, and rest labels to all other feature vectors. Since this overall strategy relies only on the visual inspection of neural signals, we believe it to be compatible with reduced availability of ground truth signals, like movement, as might be the case in locked-in participants.

### Model architecture and training

We designed a recurrent neural network in a many-to-one configuration to learn changes in HG power over sequences of 1 s (Supplementary Fig. 7). Each 128-channel HG power vector was input into a long shortterm memory (LSTM) layer with 25 hidden units for modelling sequential dependencies. From here, 2 consecutive fully-connected (FC) layers with 10 and 2 hidden units, respectively, determined probabilities of the rest or grasp class. The former utilized an eLU activation function while the latter employed softmax to output normalized probability values. In total, the architecture consisted of 17,932 trainable parameters, and was trained on a balanced dataset of rest and attempted grasping sequences by randomly downsampling from the overrepresented rest class.

We determined the model’s hyperparameters by evaluating our model’s offline accuracy using 10-fold cross-validation with data collected for training (*see* Cross-validation). For each cross-validated model, we limited training to 75 epochs during which classification accuracy of the validation fold plateaued. We used categorical cross-entropy for computing the error between true and predicted labels of each 45-sample batch and updated the weights using adaptive moment optimization (Adam optimizer)^23^. To prevent overfitting on the training data, we used a 30% dropout of weights in the LSTM and FC layers. All weights were initialized according to a He Normal distribution.^24^ The model was implemented in Python 3.8 using Keras with a TensorFlow backend (v2.8.0).

### Real-time pipeline

#### Pipeline structure

We used ezmsg, a Python-based messaging architecture (https://github.com/iscoe/ezmsg)^25^, to create a directed acyclic graph of processing units, in which all pre-processing, classification, and post-processing steps were partitioned.

#### Real-time pre-processing

Neural data was streamed in intervals of 100 ms via a ZeroMQ connection from BCI2000^20^ to our real-time pipeline, which was hosted on a separate machine dedicated to real-time inference. Incoming data updated a running 256 ms buffer, from which a 128-channel feature vector of HG power was then computed as described above (Figs. 1c and 1d). This feature vector was stored in a running buffer of 10 feature vectors (Fig. 1e), which represented 1 s of feature history for our LSTM input (Fig. 1f).

#### Classification and post-processing

A rest or grasp classification was generated every 100 ms by the FC layer, after which it entered a running buffer of classifications, which in turn was updated with each new classification. This buffer was our voting window, which contained a pre-determined number of classifications (10 and 7 for the medical communication board and the spelling interface respectively), and in which a given number of those classifications (voting threshold) were required to be grasp in order to initiate a click (Fig. 1g). This voting window and threshold were applied to prevent sporadic grasp classifications from being interpreted as an intention to execute a click. A click triggered selection of the participant’s desired row or column in the switch-scanning application (Fig. 1h).

#### Switch-scanning applications

A switch-scanning application is an augmentation and alternative communication (AAC) technology that allows users with severe motor or cognitive impairments to navigate to and select icons or letters by timing their clicks to the desired row or column during periods in which rows or columns are sequentially highlighted^26–32^. The participant generated a click by attempting a brief grasping movement as described in *Training task*.

#### Medical communication board

As a preliminary assessment of our model’s sensitivity and false positive detections, we first cued our participant to navigate to and select keys with graphical symbols from a medical communication board (Supplementary Fig. 8). Graphical symbols were obtained from https://communicationboard.io/. We used a 10-vote voting window with a 10-vote threshold (all 10 classifications within the running voting window needed to be grasp to initiate a click) and set our row and column scan rates to 1.5 per s. Finally, we enforced a lock-out period of 1 s, during which no other clicks could be produced, after clicking on a row or a button within a row (Fig. 1g). This prevented multiple clicks being produced from the same attempted grasp.

#### Spelling application

We then developed a switch-scanning spelling application, in which the participant was prompted to spell sentences (Supplementary Fig. 9). The buttons within the spelling interface were arranged in a grid design that included a center keyboard as well as autocomplete options for both letters and words. Letter and word autocompletion options were both generated by a distilBERT language model^33^ hosted on a separate server, providing inference through an API. The distilBERT model was chosen over larger language models for its faster inference speed. We added three pre-selection rows at the beginning of each switch scanning cycle as well as one pre-selection column at the beginning of column scanning cycle. These allowed the participant a brief preparation time if he desired to select the first row, or first column within a selected row. We decided to use a 7-vote voting window with a 7-vote threshold, which decreased latency from attempted grasp onset to click (*see* Click latencies) compared to when using the medical communication board. However, after several sessions of spelling and feedback from the participant, we reduced the voting threshold requirement to a 4-vote threshold (any 4/7 classifications within the running voting window needed to be grasp to initiate a click). We again enforced a lock-out period of 1 s.

#### Real-time switch-scanning

Using the communication board, the participant was instructed to navigate to and select one of the keys verbally cued by the experimenter. If the participant selected the incorrect row, the cued key was changed to be in that row. Once a key was selected, the switch-scanning cycle would start anew (Supplementary Video 1, Supplementary Fig. 8).

To test real-time spelling performance using our click detector, the participant was required to type out sentences by using the switch-scanning spelling application. The sentences were sampled from the Harvard sentence corpus^34^ and were presented at the top of the speller in faded gray text. If the participant accidentally clicked a wrong key, resulting in an incorrect letter or autocompleted word, the corresponding output text would be highlighted in red. The participant was then required to delete it using the DEL or A-DEL (auto-delete) keys respectively. Once the participant completed a sentence, he advanced to the next one by clicking the ENTER key (Supplementary Video 2, Supplementary Fig. 9). A spelling block consisted of 3–4 sentences to complete, and in each session the participant completed 1–6 spelling blocks (Fig. 2b).

### Performance evaluation

#### Sensitivity and click rates

Sensitivity was measured as the percentage of correctly detected clicks:

$$\text{Sensitivity}=\frac{N_{\text{trueclicks}}}{N_{\text{attemptedgrasps}}}\times 100\%$$

where in one session *N*_*trueclicks*_ were the total number of correct clicks and *N*_*grasps*_ were the total number of attempted grasps, and where *N*_*trueclicks*_ ≤ *N*_*attemptedgrasps*._ For a detected click to be correct (i.e., a true positive), it had to have occurred on the user interface (as visual feedback to the participant) within 1.5 s after the onset of an attempted grasp. Attempted grasps with no clicks occurring within this time period were considered false negatives. Clicks that occurred outside this time period were assumed to be unrelated to any attempted grasp and were thus considered false positives. True positive and false positive frequencies (TPF and FPF respectively) were measured per unit time and for each session were defined as the following:

$$\text{TPF}=\frac{N_{\text{TP}}}{T}=\frac{N_{\text{trueclicks}}}{T}\text{FPF}=\frac{N_{\text{FP}}}{T}$$

where *N*_*TP*_ and *N*_*FP*_ are the number of true and false positives in a session respectively, and *T* is the total spelling time for that session. Whether the participant clicked the correct or incorrect key had no bearing on sensitivity, TPF, or FPF as these metrics depended only on whether a click truly occurred following an attempted grasp.

#### Click latencies

Movement onsets and offsets were determined from the normalized pose-estimated landmark trajectories of the hand. Specifically, only the landmarks of the fingers with significant movement during the attempted grasp were considered. Then, for each attempted grasp, movement onset and offset times were visually estimated.

For each correctly detected attempted grasp, we computed both: a) the time elapsed between movement onset and algorithm detection, and b) the time elapsed between movement onset and the click appearing on the spelling application’s user interface. The latency to algorithm detection was primarily composed of the time necessary to reach the voting threshold (i.e., a 4-vote threshold usually produced at least 400 ms latency if four grasps were sequentially classified). The latency to the on-screen click appearing on the spelling interface depended on the algorithm detection latency along with additional network and computational overhead necessary for displaying the click.

#### Spelling rates

Spelling rates were measured by correct characters per minute (CCPM) and correct words per minute (CWPM). Spelled characters and words were correct if they exactly matched their positions in the prompted sentence. For example, if the participant spelled a sentence with 30 characters (5 words) with 1 character typo, only 29 characters (4 words) contributed to the CCPM (CWPM). Note that all spelling was performed with assistance of autocompletion options from the language model.

#### Cross-validation

We partitioned our training data into 10 folds such that each fold contained an equal number of rest and grasp samples of HG power feature vectors (rest samples were randomly downsampled to match the number of grasp samples). To minimize data leakage of time dependent data into the validation fold, all samples within a fold were contiguous and each sample belonged to only one fold. Each fold was used once for validation and a corresponding cross-validated model was trained on the remaining 9 folds.

#### Channel contributions and o ine classification comparisons

Using the subset of samples in the training data labeled as grasp, we computed each channel’s importance to generating a grasp classification given our model architecture. Specifically, we computed the integrated gradients from 10 cross-validated models (*see* Cross-validation) with respect to the input features from each sample labeled as grasp in the corresponding validation folds. This generated an attribution map for each sample^35^, from which we calculated the L2-norm across all 10 historical time feature vectors^2^, resulting in a 1×128 saliency vector. Due to the random initialization of weights in the RNN-FC network, models trained on features from the same set of folds were not guaranteed to converge to one set of final weights. We therefore retrained the set of 10 cross validated models 20 times and similarly recomputed the saliency vectors for each sample. The final saliency map was computed by averaging the attribution maps across all repeated samples and normalizing the resulting mean values between 0 and 1. We repeated this process using HG features from all channels except one (channel 112) and again by using features from a subset of 12 electrodes over cortical hand-knob (anatomically determined as channels 92, 93, 94, 100, 101, 102, 108, 109, 110, 116, 117, 118; Fig. 4e, Supplementary Figs. 2,3). Neither of these two model architectures were deployed for real-time BCI use.

To inform whether models trained with HG features from these smaller subsets of channels could retain robust click performance, we computed offline classification accuracies using 10-fold cross-validation (*see* Cross-validation). We repeated cross-validation (see above) such that for each of the 10 validation folds a set of 20 accuracy values was produced. We then took the average of these 20 values to obtain a final accuracy for each fold. For each subset of channels, a confusion matrix was generated using the true and predicted labels across all validation folds and all repetitions.

### Statistics and Reproducibility

#### Statistical analysis

Spelling blocks with a specific voting threshold were collected on no more than nine sessions. Given this small sample size, we could not assume normality in the distribution of the sample mean of any of the performance metrics (sensitivity, TPF, FPF, latencies, CCPM, CWPM). Therefore, we decided to use the non-parametric Wilcoxon Rank-Sum test to determine whether there were significant differences between performance metrics from spelling blocks where different voting thresholds were applied. A P-value less than 0.05 was considered significant. Similarly, we used the Wilcoxon Rank-Sum test to determine whether there were significant differences in offline classification accuracies when different configurations of channels were used from model-training and validation. We additionally used a Holm-Bonferroni correction to adjust for multiple comparisons.

#### Reproducibility of experiments

Neural data collection and processing as well as decoder performance were reproducible across sessions as the participant was able to repeatedly demonstrate click control using neural signals from attempted hand movements to spell sentences. However, as this study reports on the first and only participant in this trial so far, further work will be necessary to test the reproducibility of these results in other participants.

## Results

### Long-term usage with a fixed click detector

The participant used the fixed click detector to effectively control a switch-scanning application for a total of 626 min spanning a 90-day period that started on Day 21 after the completion of training data collection (Fig. 2b). Specifically, we recorded one session with the medical communication board and 17 sessions with the spelling application. We defined Day 0 as the last session of training data collection. We used a voting threshold of 10/10 votes with the communication board. Using the spelling application, we initially used a voting threshold of 7/7 votes, but reduced this threshold to 4/7 votes on Day + 81 as the participant reported that he preferred an increased sensitivity despite the resulting increase in false positive detections. We found that the decoder performance remained robust for 111 days.

### Switch-scanning performance

With the switch-scanning medical communication board, the click-detection model achieved 93% sensitivity (percentage of detected clicks per attempted grasps) with a median latency of 1.23 s from movement onset to on-screen click (visual feedback on the user interface) using a 10-vote threshold. No false positives were detected.

**Figure 3:**
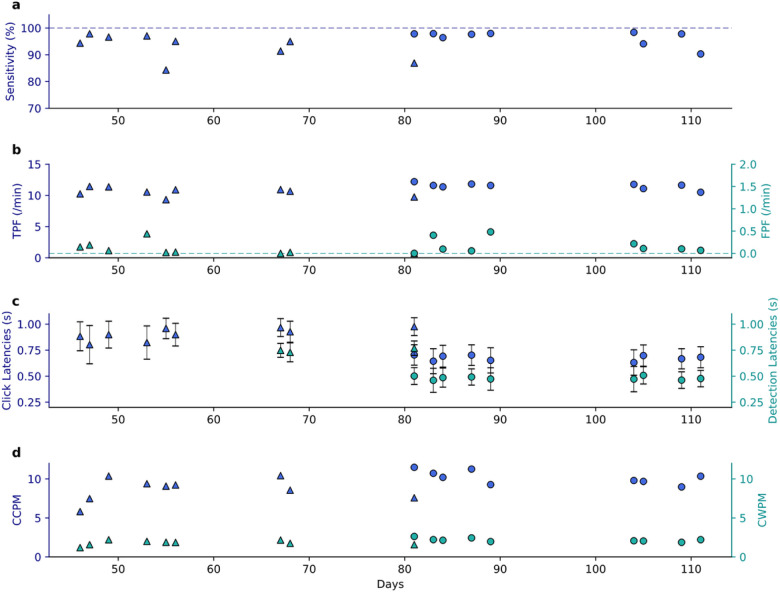
Long-term switch-scanning spelling performance. Across all subplots, triangular and circular markers represent metrics using a 7-vote and 4-vote voting threshold respectively. **(a)** Sensitivity of grasp detection for each session. Dashed line delineates 100% sensitivity. **(b)** True-positive and false-positive frequencies (TPF and FPF) measured as detections per minute. Dashed line delineates 0 FPF. **(c)** Average latencies with standard deviation error bars of grasp onset to algorithm detection and to on-screen click. The averages and standard deviations were computed from latency measurements across all spelling blocks from one session using the same voting threshold. Using 7-vote and 4-vote voting thresholds, onscreen clicks happened an average of 207 ms and 203 ms respectively after detection. Note that detection latencies were not registered in the first six sessions. **(d)** Correct characters and words per minute (CCPM and CWPM).

Using the switch-scanning spelling application (from Day + 46 to Day + 111), the click detector achieved a median detection sensitivity of 94.9% using a 7-vote threshold, and a sensitivity of 97.8% when using a 4-vote threshold (*P = 0.057*, Wilcoxon Rank-Sum test; Fig. 3a). The median true positive frequency (TPF) was 10.7 per min using a 7-vote threshold, which improved to 11.6 per min when using a 4-vote threshold (*P = 0.005*, Wilcoxon Rank-Sum test; Fig. 3b); the median false positive frequency (FPF) was 0.029 per min (1.74 per h) using a 7-vote threshold and 0.101 per min (6.03 per h) when using a 4-vote threshold (*P = 0.20*, Wilcoxon Rank-Sum test; Fig. 3b).

As expected, we observed a decrease in latency from movement onset to algorithmic detection and onscreen click when switching from the 7-vote to the 4-vote threshold (Fig. 3c). Using the 7-vote threshold, the median detection latency was 0.75 s and significantly dropped to 0.48 s using the 4-vote threshold (*P = 0.013*, Wilcoxon Rank-Sum test). Meanwhile the median on-screen click latency was 0.93 s using the 7-vote threshold and dropped to 0.68 s using the 4-vote threshold (*P = 3 × 10*^*−4*^, Wilcoxon Rank-Sum test). The delay between algorithmic detection and on-screen click was consistently ~ 200 msec, due to network and computational overhead.

Consequently, the participant was able to achieve high rates of spelling (Fig. 3d). Specifically, median spelling rate was 9.1 correct characters per minute (CCPM) using the 7-vote threshold, which significantly improved to 10.2 CCPM using the 4-vote threshold (*P = 0.031*, Wilcoxon Rank-Sum test). Similarly, he achieved 1.85 correct words per minute (CWPM) using the 7-vote threshold, which significantly improved to 2.14 CWPM using the 4-vote threshold (P = 0.015, Wilcoxon Rank-Sum test). In one session, the participant achieved a spelling rate greater than 11 CCPM with the 4-vote threshold, which to our knowledge is the highest spelling rate achieved using single-command BCI control with a switch-scanning spelling paradigm.

#### Decoder retraining due to transient performance drop

On Day + 118 (Supplementary Fig. 10 for timeline), the detector sensitivity fell below the pre-set performance threshold of 80% (Supplementary Fig. 11), which was likely due to a drop in the movementaligned HG response across a subset of channels (Supplementary Fig. 12). We found no hardware or software causes for the observed deviations in HG responses. Moreover, the participant had no subjective change in strength, no changes on detailed neurological examination or cognitive testing, and no new findings on brain computerized tomography images.

To ensure that BCI performance was not permanently affected, we retrained and tested a click detector with the same model architecture using data collected roughly four months after the observed performance drop (Supplementary Note 5). The new click detection algorithm used a total of 15 min of training data, which was all collected within one day (Supplementary Fig. 13a); afterward, the model weights remained fixed again. To determine the optimal voting threshold for continued long-term use, we additionally evaluated real-time click performance using all voting thresholds from 2/7 to 7/7 votes with this new click detection algorithm (Supplementary Fig. 14).

The participant used this retrained click detector for a total of 428 min in six sessions spanning a 21-day period after re-training (Supplementary Fig. 13b). The optimal combination of sensitivity (Supplementary Note 6) and false detections was achieved using a 6-vote threshold. Using this threshold, we achieved similar performance metrics to those from the original click detector with a 4-vote threshold, namely a median detection sensitivity of 94.8%, median TPF and FPF of 11.3 per min and 0.20 per min respectively, and a median CCPM and CWPM of 10.1 and 2.2 respectively (for all comparisons *P* > 0.05, Wilcoxon Rank-Sum test) (Supplementary Fig. 15). Expectedly the median on-screen click latency was 0.86 s, roughly 200 ms higher compared to the previous 4-vote threshold, due to the two extra votes required for generating a click (*P = 10*^−3^, Wilcoxon Rank-Sum test).

#### Electrode contributions to grasp classification

**Figure 4:**
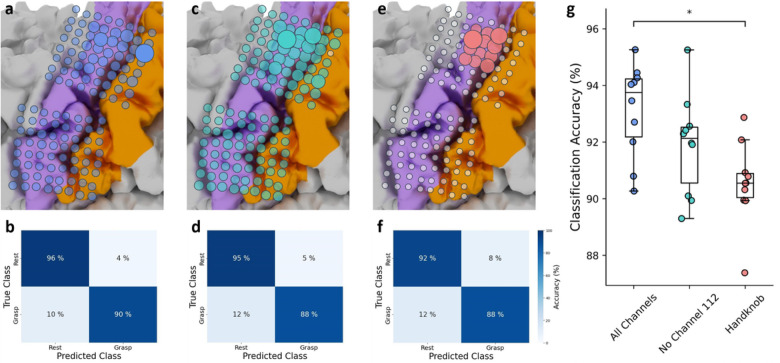
Channel importance for grasp classification. Saliency maps for the model used in real-time, a model using HG features from all channels except from channel 112, and a model using HG features only from channels covering cortical hand-knob are shown in **(a)**, **(c)** and **(e)** respectively. Electrodes overlayed with larger circles represent greater importance for grasp classification. White and transparent circles represent electrodes which were not used for model training. Mean confusion matrices from repeated 10-fold CV using models trained on HG features from all channels, all channels except for channel 112, and channels covering only the cortical hand-knob are shown in **(b)**, **(d)**, and **(f)** respectively. For all confusion matrices, the percent value in each element of the matrix represents how many times the validation features across all repetitions of all validation folds were predicted correctly or incorrectly. The mean classification accuracy was computed from averaging the values on the diagonal of the confusion matrix. **(g)** Box and whisker plot showing the offline classification accuracies from 10 cross-validated testing folds using models with the above-mentioned channel subsets. Specifically, for one model configuration, each dot represents the average accuracy of the same validation fold across 20 repetitions of 10-fold CV (see Methods: Channel contributions). Offline classification accuracies from CV-models trained on all features from all channels were statistically higher than CV-models trained on features from channels only over cortical hand-knob (*P = 0.015, Wilcoxon Rank-Sum test with 3-way Bonferroni-Holm correction). Offline classification accuracies from CV-models trained on features from all channels except for channel 112 were not statistically different from those trained on features from all channels or features only from channels only over cortical hand-knob.

To assess which channels produced the most important HG features for classification of attempted grasp, we generated a saliency map across all channels used to train our original model (Fig. 4a). As expected, channels covering cortical face region were generally not salient for grasp classification. The channel producing the most salient HG features was located in the upper-limb area of somatosensory cortex (channel 112, Supplementary Fig. 16), with a saliency value 55% and 88% higher than the next two most salient channels respectively (Supplementary Fig. 16a). Indeed, prior to the observed performance drop, this channel had a relatively amplified spectral response compared to other channels during attempted grasp. We then computed the corresponding offine classification accuracy of our original model architecture for comparison to a model architecture without channel 112 and an architecture using channels only over cortical hand-knob (*see* Methods: Channel contributions and offline classification comparisons); the mean accuracy from repeated 10-fold cross-validation (CV) was 92.9% (Fig. 4b).

To ensure that real-time classification accuracy was not entirely driven by channel 112, we evaluated a model trained on HG features from all other channels offline. As expected, this model relied strongly on channels covering the cortical hand-knob region (Fig. 4c), and notably was not as dependent on a single channel; the saliency of the most important channel was only 23% and 60% larger than the next two most salient channels, respectively (Supplementary Fig. 16b). The offline mean classification accuracy from repeated 10-fold CV was 91.7% (Fig. 4d), which was not significantly lower compared to the mean accuracy using all channels (*P = 0.139*, Wilcoxon Rank-Sum test with 3-way Bonferroni-Holm correction, Fig. 4g).

As channels covering the cortical hand-knob region made relatively larger contributions to decoding results, we investigated the classification accuracy of a model trained on HG features from a subset of electrodes covering only this region (Fig. 4e). Saliency values followed a flatter distribution; the saliency of the most important channel was only 21% and 44% larger than the next two most salient channels respectively (Supplementary Fig. 16c). Though the offline mean classification accuracy from repeated 10-fold CV remained high at 90.4% (Fig. 4f), it was statistically lower compared to the mean accuracy using all channels (*P = 0.015*, Wilcoxon Rank-Sum test with 3-way Bonferroni-Holm correction, Fig. 4g). This suggests that a model trained on HG features from only the cortical hand-knob could still produce effective click detection, but parameters used for data labeling, model training, and post-processing may need to be more thoroughly explored to optimize click performance.

## Discussion

In this study we show that a clinical trial participant with ALS was able to use a fixed decoder trained on a limited multichannel electrocorticographic (ECoG) dataset to generate stable real-time clicks over a period of three months. Specifically, the participant used his click detector to select the appropriate letters and words to form sentences using a switch-scanning spelling application. Our detector’s high sensitivity (97.8%), low false positive frequency (0.101 per min) and minimal latency between onset of attempted grasp and click (0.48 s) allowed him to quickly and reliably spell sentences over a several months without retraining the model.

A significant barrier to the use of BCI systems by clinical populations outside of the laboratory is that users must often undergo an extensive period of training for optimizing fixed decoders^1^, or daily model retraining or updating^3^. For example, reliable switch-scan spelling was demonstrated for up to 36 months using a fixed decoder but required several months of data collection to optimize parameters for inhibiting unintentional brain clicks^1,17^. However, our click detector’s long-term performance with a relatively small training dataset suggests a potentially reduced need for model optimization using ECoG signals with higher spatial density (for example, 12 electrodes with 4 mm pitch covering the cortical hand-knob region in this study compared to 4 electrodes with 10 mm pitch in the aforementioned one). Similarly, an endovascular electrode stent-array was recently used to train an attempted movement detector^4^. Though this is an extremely promising BCI technology for click decoding, the anatomical constraints on the number and proximity of electrodes in the stent-array to motor cortex may make it difficult to scale up from simple brain-clicks to more complex BCI commands^3,36,37^. The device we used in this study may have included more electrodes over upper-limb cortex than was necessary for click detection, but it allowed us to explore the upper bounds of click performance that might be expected for a device with these capabilities in a participant with ALS.

Our model detected intended clicks with high sensitivity and low false positive rates. The high sensitivity was likely attributable to the high contrast between HG power during movement vs. rest, or baseline conditions, which had previously enabled real-time grasp detection^3,38,39^ but may have not been as robustly detected by the Activa PC + S device^40^ in previous work^1^. The voting window provided a simple yet effective heuristic strategy for inhibiting false detections; post-hoc analysis of real-time performance revealed that false detections particularly increased when less than three votes were required for producing a click (Supplementary Fig. 13). We initially chose a conservative voting threshold of 100% (7/7 votes), but later adjusted it to 57% (4/7 votes), as the participant reported that he preferred an increased sensitivity and reduced click latency despite a slight increase in false detections. This experience supports the utility of allowing users to fine-tune algorithmic parameters that can affect BCI performance and the user experience and that may vary significantly among users and among different applications that use a click-detector.

Using a switch-scanning spelling application, the participant achieved high spelling rates by timing his clicks to select the appropriate row or column. Our results improve upon the previous work by Vansteensel et al. (2016) in which a participant with ALS was implanted with four contacts over hand motor cortex and achieved a spelling rate of 1.8 CPM and a latency of 1 s per click. These results may have been limited by lower sensitivity for high frequency activity, a single bipolar channel, and a 5 Hz transmission rate of power values (related to energy consumption of wireless signal transmission, see Vansteensel et al., 2016). In fact, our spelling rates were comparable to those from other clinical populations who have used switch scanning keyboards without a BCI, including people living with ALS^41^ or other causes of motor impairments^42^. It is worth noting that although the integration of eye-tracking with click decoding may enable even faster user interface navigation and spelling rates^4,43^, it may also cause eyestrain during long periods of use^44^ and worsen as residual eye movements deteriorate in latestage ALS^45–47^.

To explore whether more limited electrode coverage of sensorimotor cortex would be sufficient for comparable click performance, we conducted a channel-wise saliency analysis. Despite the substantially higher saliency of one channel in post-central gyrus adjacent to the cortical hand knob, many of our highly salient channels were located over the pre-central gyrus at the cortical hand knob^11,48^, and a virtual grid confined to this area had only a slight reduction in grasp classification accuracy (90.4%, vs. 92.9% in the all-channel model). As suggested by our high offline accuracy, a click detector of comparable performance might be effective using this smaller cortical coverage while leaving open the possibility of training models for multiclass or cursor-based control.

After nearly four months without re-training or updating our model, we observed a drop in BCI performance caused by a modest decrease in the modulation of upper-limb HG power in several electrodes over hand area of sensorimotor cortex. This decrease was especially pronounced in the most salient channel used to train the original detector, so it was not unexpected that BCI performance was affected. There were no accompanying new neurological symptoms or changes in cognitive testing, nor any evidence of adverse events or device malfunction. Variations in signal amplitude and spectral energy similar to those we observed in our participant have been reported in ECoG signals recorded for several years by the Neuropace (TM) RNS system^49^. However, the RNS system typically stores samples of ECoG from only 4 bipolar channels (8 contacts) in each patient, and is indicated for patients with epilepsy, not patients with ALS, for which there is scarce data on long-term ECoG. We are aware of only one such study^17^, but this study did not report signal characteristics on the granular timescale necessary for comparison to our results. Regardless of the cause, our click detector’s small amount of training data did not include the signal regime we observed during the performance drop. Nevertheless, we successfully tested another click detector, which was retrained with even less data using a similar workflow, and achieved equally robust performance in subsequent testing sessions, suggesting that long-term discernability of HG activity was not affected. In the future, it may be possible to achieve both high performance and longevity by updating our model periodically, for example once every few months, simulating a periodic in-lab or outpatient checkup.

Our study adds to the expanding literature on ECoG as an effective recording modality for long-term BCI use. Importantly, due to the participant’s residual upper limb movement, we were able to assess click performance using his “ground-truth” movement attempts. However, more work is needed to determine how a rapidly trained fixed click detector can provide long-term efficacy for the population of individuals suffering from more severe movement impairments. Robust click-detection capability complements recent major advancements in real-time spelling^2,50^ and speech decoding^51,52^ and provides a more application-agnostic capability for navigating menus and applications. Optimal spelling performance, however, was likely not realized as the linguistic statistics of our Harvard sentence prompts were not sufficiently representative of the word sequences on which our language model was trained. Therefore, we expect that spelling rates could be substantially improved during free-form spelling and even more so with a language model tuned to the linguistic preferences of the participant. Further, there is likely a userspecific regularity of model updates that would optimize the balance between independent long-term BCI use and technician intervention, which is especially relevant during home-use. Finally, we expect that click detectors, in addition to their utility as a communication tool, may be critical for accessibility software beyond spelling interfaces or communication boards such as web-browsers, internet of things (IoT), and multimedia platforms, and thus merit further investigation.

## Data Availability

Source data for Figs. 2–4 can be accessed in Supplementary Data 1 (accessible upon manuscript acceptance). Beginning immediately after publication, individual participant data (neural, and behavioral) and study protocol will be available from the corresponding author upon reasonable request.
